# Assessing the Perception of Pharmacy Students on Launching a Doctor of Pharmacy/Master of Public Health Dual Degree Program in Saudi Arabia: A Multi-Institutional Cross-Sectional Study

**DOI:** 10.3390/ijerph19138014

**Published:** 2022-06-30

**Authors:** Hisham A. Badreldin, Khalid Bin Saleh, Aisha F. Badr, Abdullah Alhifany, Shuroug A. Alowais, Sumaya N. Almohareb, Nada Alsuhebany, Abdulmajeed Alshehri, Mohammed Alzahrani, Anas Aldwsari, Ohoud Aljuhani, Ghazwa B. Korayem, Khalid Al Sulaiman, Allulu Alturki, Hayfa Alhaidal, Yazeed Ghawaa

**Affiliations:** 1 King Abdullah International Medical Research Center, Department of Pharmacy Practice, College of Pharmacy, King Saud Bin Abdulaziz University for Health Sciences, Riyadh 11481, Saudi Arabia; binsalehkh@gmail.com (K.B.S.); owaiss@ksau-hs.edu.sa (S.A.A.); moharebsu@ksau-hs.edu.sa (S.N.A.); suhebanyn@ksau-hs.edu.sa (N.A.); abdulmajeed483@gmail.com (A.A.); zahranim@uic.edu (M.A.); aldawsari095@ksau-hs.edu.sa (A.A.); alsulaimankh@hotmail.com (K.A.S.); alturkkia336@ksau-hs.edu.sa (A.A.); alhaidal086@ksau-hs.edu.sa (H.A.); 2 Department of Pharmacy Practice, Faculty of Pharmacy, King Abdulaziz University, Jeddah 21589, Saudi Arabia; afbadr@kau.edu.sa (A.F.B.); oaljuhani@kau.edu.sa (O.A.); 3 Department of Clinical Pharmacy, College of Pharmacy, Umm AlQura University, Makkah 21955, Saudi Arabia; aahifany@uqu.edu.sa; 4 Department of Pharmacy Practice, College of Pharmacy, Princess Nourah bint Abdulrahman University, Riyadh 11481, Saudi Arabia; ghazwa.krayem@gmail.com; 5 Saudi Critical Care Pharmacy Research (SCAPE) Platform, Riyadh 11481, Saudi Arabia; 6 Department of Clinical Pharmacy, College of Pharmacy, King Saud University, Riyadh 11481, Saudi Arabia; yghawaa@ksu.edu.sa

**Keywords:** pharmacy, pharmacy students, education, public health, dual degree, Saudi Arabia

## Abstract

There is a lack of Doctor of Pharmacy (PharmD) and Master of Public Health (MPH) dual degree programs in Saudi Arabia. This study aims to examine current pharmacy students’ perceptions regarding establishing such a program and the perceived limitations and advantages of pursuing such a degree. We conducted a cross-sectional web-based short survey to assess the feasibility of establishing a PharmD/MPH dual degree program in several randomly selected pharmacy schools in Saudi Arabia. Our cohort consisted of 657 students. Almost 56% were males, and nearly 58% were fourth-year pharmacy students. Close to 85% had a “very well” or “well” understanding regarding the pharmacist’s role in the public health area, and almost 70% stated that they see themselves playing a role in public health as a future pharmacist. Nearly 93% reported that they are either “very likely” or “likely” to enroll in such a program if given the opportunity. Almost 80% felt it would increase their job opportunities. On the other hand, close to 70% felt it would increase workload and stress. This study highlights pharmacy students’ positive perceptions regarding establishing a PharmD/MPH dual degree program in Saudi Arabia. The study results could be utilized as the starting point to propose and establish this program to health education policymakers in Saudi Arabia.

## 1. Introduction

The profound impacts of pharmacists on public health are evident and have been acknowledged by many professional organizations [1,2,3]. In addition to their essential roles in chronic disease management and prevention, pharmacists are involved in providing many public health roles, which include chronic disease care, immunizations, mental health screening, and preventive services, apart from being medication experts [4,5,6,7,8]. Also, pharmacists are easily accessible in community settings that give them the opportunity to counsel patients and optimize medication use, as well as to have more substantial roles in public health either at a macro-level, which target the whole population, or at a micro-level, targeting specific patients [9,10]. 

The expansion of the pharmacy’s professional scope is ongoing and has encouraged many professional organizations to promote the integration of public health education into the Doctor of Pharmacy (PharmD) curriculum to prepare students for future public health roles [11,12]. In response to pharmacists’ growing needs in public health services, schools of pharmacy incorporated public health education into the curricula, which was mandated by the Accreditation Council for Pharmacy Education (ACPE) as a required element of the didactic Doctor of Pharmacy curriculum. In addition, public health topics such as epidemiology, disease prevention, and health promotion were considered essential components of the Center for the Advancement of Pharmaceutical Education (CAPE) Educational Outcomes, which is an initiative that was developed by the American Association of Colleges of Pharmacy (AACP) to improve pharmacy curriculum at pharmacy schools, and which has led to incorporating public health courses in pharmacy schools’ curricula [13]. 

As a result of these recommendations, public health courses were implemented in pharmacy schools that allow students to be exposed to the basic concepts of public health without in-depth education [2]. However, the development of joint PharmD/Master of Public Health (MPH) programs was encouraged by the American Public Health Association, which led pharmacy schools to offer this joint PharmD/MPH programs to provide more exceptional public health education for students to prepare them for future public health roles. In the United States, there are 23 schools of pharmacy in 2015 that offer dual PharmD/MPH programs, and the prevalence is increasing in order to meet the expansion of pharmacists’ roles in public health [14,15]. With the rapid emergence of dual degree programs, pharmacy students are aware of the importance of these programs and have been interested in pursuing them to meet future job demands [14,15,16]. 

Until 2020, Saudi Arabia has had close to 30 schools of pharmacy in which the majority of them are offering the Doctor of Pharmacy Degree. The structure of the curriculum is similar to the one currently adopted in the US. Saudi Arabia is evolving drastically in all aspects, including health and education, in alignment with the Vision of 2030. Before establishing such a program by the Saudi Ministry of Education, the level of interest for the proposed dual degree program should be explored among pharmacy students to evaluate potential interest and enrollment. In response to this growing perspective, we aim to investigate the perception of pharmacy students on launching a PharmD/MPH dual degree program in Saudi Arabia.

## 2. Materials and Methods

We conducted a cross-sectional web-based short survey to assess the feasibility of establishing a PharmD/MPH dual degree program in Saudi Arabia. A seven-question survey was distributed to all students enrolled in the PharmD program in several pharmacy schools in Saudi Arabia. A survey was sent to each student’s official school email. Saudi Arabia currently has 30 pharmacy schools. For the purpose of this study, eight schools with different class sizes and different geographical locations were randomly selected. The questionnaire was adopted with permission in English language from a previously utilized questionnaire in a similar study conducted by Holtzman CW et al. study [14]. The objective of this survey was to gather initial information in order to assess the feasibility of developing a PharmD/MPH dual degree program. This study was approved by the Institutional Review Board at King Abdullah International Medical Research Center. 

The first question is intended to explore if pharmacy students understand the role of the pharmacist in public health. The second question intended to examine if pharmacy students see themselves playing a role in public health as future pharmacists. The third question intended to explore pharmacy students’ level of interest in being involved with public health activities as a pharmacist. The fourth question intended to explore the likelihood of enrolling in such a program if given the opportunity. The fifth question intended to explore whether such program might attract future pharmacy students. The sixth question intended to explore the perceived advantages to a dual PharmD/MPH degree. The seventh question intended to explore the perceived limitations of a dual PharmD/MPH degree in which students were allowed to choose one or more options. 

We used descriptive statistics to analyze the data in which continuous variables were presented as means, standard deviations, or medians and interquartile ranges where appropriate. Categorical data were presented as frequency and percentages. The collected data were compiled using Microsoft Excel 2010 (Office 365, Microsoft Ltd., Redmond, WA, USA) and analyzed by using Statistical Package for Social Sciences 20.0 version (SPSS Inc. Chicago, IL, USA).

## 3. Results

### 3.1. Baseline Characteristics 

A total of 657 out of 1000 students completed the survey, indicating a 65.7% response rate. Of the included students, almost 56% were males, and 44% were female. Nearly 58% were fourth-year pharmacy students. The average age of the included cohort was 22 years old. As stated previously, Saudi Arabia has 30 public and private pharmacy schools, a pool from which we randomly selected eight to receive our questionnaire. Table 1 highlights the detailed characteristic information of the pharmacy students included.

### 3.2. Perception and Likelihood of Pharmacy Students to Peruse a Dual PharmD/MPH Degree

When asked “how well you think you understand the role of the pharmacist in the public health area”, nearly 85% had a “very well” or “well” understanding of the pharmacist role in the public health area. Almost 70% stated that they see themselves playing a role in public health as a future pharmacist. Close to 97% stated that they are either “somewhat interested” or “very interested” in being involved with public health activities as a pharmacist. When asked about the likelihood of enrolling in such a program, close to 93% stated that they are either “very likely” or ‘’likely’’ to enroll in such a program if given the opportunity. Finally, approximately 85% reported that such a program might be attractive to future pharmacy students, as shown in Table 2.

### 3.3. Perceived Advantages and Limitation to Pursue a PharmD/MPH Dual Degree

**Figure 1:**
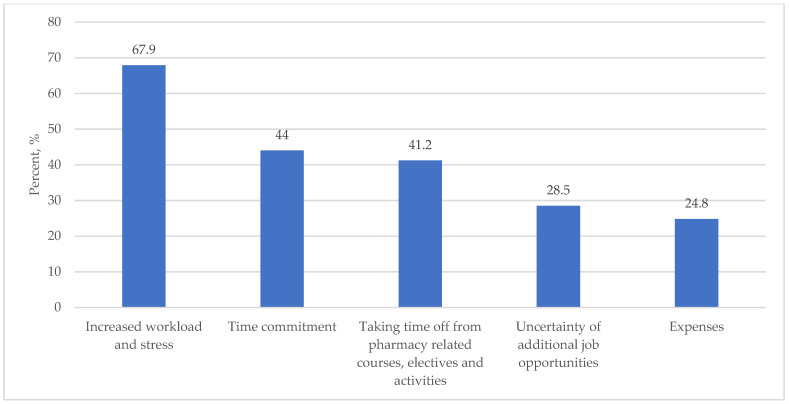
Perceived advantages in pursuing a PharmD/MPH Dual Degree.

**Figure 2:**
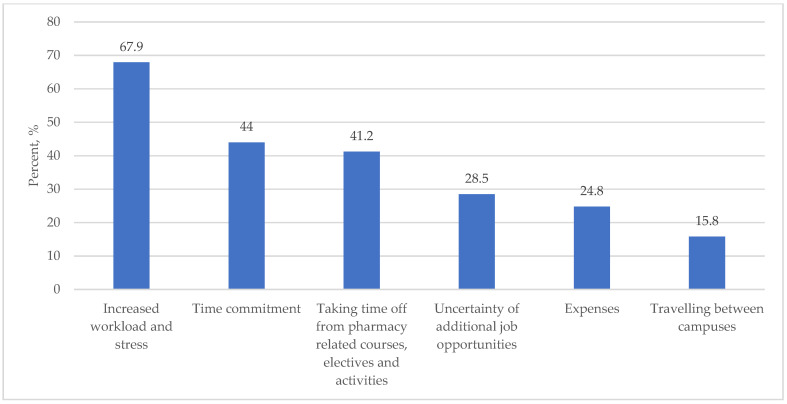
Perceived limitations in pursuing a PharmD/MPH Dual Degree.

For the perceived advantages of pursuing a PharmD/MPH dual degree, 80.1%, 71.8%, and 70.6% felt it would increase their job opportunities, increase the ability to serve patients and community, and increase their knowledge and understanding of public health concepts, respectively. Other perceived advantages are shown in Figure 1. In terms of the perceived limitations to pursue a PharmD/MPH dual degree, 67.9%, 44.0%, and 41.2% felt it would increase workload and stress, increasing the commitment, followed by taking time away from pharmacy-related courses, electives, and activities, respectively. Other perceived limitations are shown in Figure 2.

## 4. Discussion

The public’s perception of the pharmacist’s role continues to expand by increasing pharmacist involvement in the community, as well as through increased patient interaction. Nutritionists, nurses, and physicians are recognized professions within the public health workforce, yet the pharmacist public health role is not clearly described nor promoted. The aim of this study was, therefore, to run a feasibility analysis to evaluate the perception and attitude of Saudi pharmacy students toward establishing the Doctor of Pharmacy/Master of Public Health dual degree. 

Of the randomly selected institutions, we had a 65.7% response rate with an overall good variation of male (56%) and female (44%) respondents. The majority (84.8%) of students thought they understood the pharmacist’s role in public health “very well”, and for “well”, most (68.6%) see themselves playing a role in public health in the future. Almost all surveyed students (97.1) were very or somewhat interested in having a public health involvement in their pharmacy career, and only 6.8% stated that they are not likely to be enrolled in a PharmD/MPH dual program. Only 1.1% did not think such a program would be attractive to future pharmacy students. Moreover, perceived advantages of such a program were mainly for having increased job opportunities (80.1%), followed by an increased ability to serve patients (71.8%) and the community, and finally, increased knowledge and an understanding of public health concepts (70.6%), whereas perceived limitations mainly concerned an increased workload and additional stress (67.9%). Overall, the findings of this study are very similar to that of previous feasibility studies done by Holtzman et al., suggesting a willingness, preparedness, and enthusiasm of adding this dual degree of PharmD/MPH to pharmacy schools [14,15]. Assessing the perception of key stakeholders for the development of such a program can be considered for future studies at the international level, which may help in comparing the different viewpoints for pharmacy students at the global level.

Although this study had the limitation of a small sample size, it was multi-centered across the kingdom and provides an insight of pharmacy students on this type of degree. One major limitation to the study is not assessing their knowledge on public health rather than having them subjectively answering how well they know of the pharmacist’s role in public health. This survey provides an insight on how pharmacy students in general are willing to expand their role to better serve their community. We believe that establishing such a program in Saudi Arabia is justified to expand the pharmacist’s essential roles as patients move through the continuum of care. In addition, we believe that such programs will empower pharmacists to have ample opportunity to provide population-based care such as chronic disease management and prevention, health planning, informing, and improving quality of care, immunization delivery, noncommunicable diseases screening, preventive services, as well as many other opportunities [17,18]. In the future, we plan to assess other national stakeholders’ perceptions regarding establishing such a program in Saudi Arabia. 

## 5. Conclusions

Given the potential expansion of the pharmacist’s role in Saudi Arabia, as well as pharmacy students’ preparedness and willingness to actively participate in public health, the addition of a dual PharmD/MPH degree further refines pharmacists’ roles in our community. Enriching the pharmacy curriculum with the option of this dual degree would fulfill the pharmacist’s expanded responsibilities in order to control costs and improve the overall quality of care.

## Figures and Tables

**Table 1:** Included student characteristics.

Characteristics	*n* = 657
**Sex, *n* (%)**	
Female	287 (43.7)
Male	370 (56.3)
**Age in years, mean (SD)**	22.2 (1.2)
**Academic year, *n* (%)**	
First academic year	0 (0.0)
Second academic year	27 (4.1)
Third academic year	63 (9.6)
Fourth academic year	380 (57.8)
Fifth academic year	95 (14.5)
Sixth academic year	92 (14.0)
**Academic Institution, *n* (%)**	
KSU	147 (22.4)
KSAU-HS	59 (9.0)
PNU	87 (13.2)
UQU	171 (26.0)
TU	53 (8.1)
KAU	132 (20.1)
Others	8 (1.2)

Abbreviations: KAU, King Abdulaziz University; KSU, King Saud University; KSAU-HS, King Saud bin Abdulaziz University for Health Sciences; PNU, Princess Nourah Bint Abdulrahman University; TU, Taif University; UQU, Umm Al Qura University; Others: Including Al-Jouf University and Shaqra University.

**Table 2:** The perception and likelihood of pharmacy students regarding a dual PharmD/MPH degree.

Student’s Perceptions Questions, *n* (%)	*n* = 657
**I think I understand the role of the pharmacist in the public health area? *n* (%)**	
Very Well	171 (26.0)
Well	386 (58.8)
Not well at all	100 (15.2)
**I see myself playing a role in public health as a future pharmacist, *n* (%)**	
Yes	451 (68.6)
No	34 (5.2)
Not sure	172 (26.2)
**Please rate your level of interest in being involved with public health activities as a pharmacist, *n* (%)**	
Very interested	334 (50.8)
Somewhat interested	304 (46.3)
Not interested or don’t know	19 (2.9)
**How likely would you consider enrolling in such a program if given the opportunity to obtain a dual PharmD/MPH degree, *n* (%)**	
Very likely	444 (67.6)
Likely	168 (25.6)
Not likely	45 (6.8)
**I consider such a program attractive to future pharmacy students, *n* (%)**	
Yes	558 (84.9)
No	7 (1.1)
Not sure	92 (14.0)

## Data Availability

Not applicable.
